# Factors associated with worsened clinical symptoms of psoriasis and disease-related quality of life during the COVID-19 lockdown: A cross-sectional study

**DOI:** 10.3389/fmed.2022.1027853

**Published:** 2023-01-10

**Authors:** Julius Burkauskas, Margarita Slabadiene, Aurelija Podlipskyte, Vesta Steibliene

**Affiliations:** ^1^Laboratory of Behavioral Medicine, Neuroscience Institute, Lithuanian University of Health Sciences, Palanga, Lithuania; ^2^Psychiatry Clinic, Lithuanian University of Health Sciences, Kaunas, Lithuania

**Keywords:** COVID-19, depression, psoriasis, quality of life, stress psychological

## Abstract

**Objective:**

In this cross-sectional study, we aimed to evaluate the factors associated with psoriasis symptom worsening and impaired quality of life (QoL) in individuals with psoriasis during the COVID-19 pandemic lockdown.

**Methods:**

During the second COVID-19 national lockdown (January–April 2021) in Lithuania, individuals diagnosed with psoriasis were invited to fill in an anonymous online survey including sociodemographic and life-style factors, psoriasis-related clinical symptoms, the Dermatology Life Quality Index (DLQI) and the Patients' Health Questionnaire (PHQ).

**Results:**

A total of 297 respondents completed the survey. The majority of them (52.5%) reported worsened clinical symptoms of psoriasis during the COVID-19 lockdown period. In total, 43.1% of responders reported significant depressive symptoms (PHQ-9 ≥ 10) and 23.6% reported impaired disease-related QoL (DLQI > 10). The strongest predictor of psoriasis symptoms worsening was the need for changes in psoriasis treatment, with an odds ratio (OR) of 2.73 (95% CI 1.37–5.44, *p* = 0.004) and decreased income (OR = 2.33, 95% CI 1.30–4.17, *p* = 0.004). The strongest predictor of impaired QoL was male sex (OR = 3.35, 95% CI 1.70–6.59, p < 0.001). Contribution of specific depressive symptoms was evident for both models.

**Conclusion:**

Worsening of psoriasis symptoms during the COVID-19 lockdown was associated with decreased income, psoriasis treatment changes and depression symptoms. Impaired QoL was associated with male sex, symptom worsening and depression. Specific depression symptoms may have contributed to more symptom worsening and impaired QoL than the depressive symptomatology as a whole.

## Introduction

Coronavirus disease 2019 (COVID-19) outbreak, with lockdown periods and strict quarantine requirements, and the fear of being infected and transmitting the disease, led to significant life style changes, that were associated with psychological distress and the risk of deterioration of mental health (1, 2). During the COVID-19 lockdowns, a considerable number of subjects with somatic illnesses may have found it more difficult to access health care services and receive their usual treatment, leading to deterioration of clinical symptoms (3–5). Overall, the COVID-19 pandemic lockdown has affected many people with somatic conditions, as well as patients' psychological wellbeing and quality of life (QoL) (6); individuals with psoriasis were no exception (7).

Psoriasis is a chronic, papulosquamous, multisystem inflammatory skin disease (8) with a prevalence varying globally from 0.5 to 11.4% and affecting over 125 million people worldwide (9, 10). Psoriasis is known to be associated with multiple metabolic, arthritic and cardiovascular comorbidities (11), seriously diminishes patients' QoL (12), and might be regarded as life-altering and stigmatizing (13). There are significant correlations between psychological distress and clinical severity of psoriasis symptoms (14, 15). Risk factors, such as stress, lifestyle changes, smoking, alcohol use and mental distress symptoms such as depression and anxiety could trigger psoriasis onset and also contribute to the need for prolonged treatment, or even cause treatment resistance (16–19).

Psoriasis is a benign disease, and as such does not affect patients' survival; however, it has a profound impact on individuals' disease-related QoL (20). Previous studies have shown that factors associated with impaired psoriasis-related QoL are longer duration of the disease, specific somatic symptoms such as itch and pain, which lead to worse physical functioning. However, in some studies, QoL was unrelated to disease severity; the strongest relationships with QoL were found for disease perception and stress coping habits (21–24). However, it is still unclear how these factors might have contributed to disease symptom worsening and impaired QoL during the COVID-19 pandemic.

The COVID-19 pandemic posed as an additional stressor for individuals with psoriasis, and a number of factors associated with the burden of the pandemic may have contributed to the worsening of disease symptoms and impaired QoL. For example, changes in family status during the COVID-19 pandemic may have affected individuals' ability to cope with stress, as having a significant other may have acted as a form of support during difficult times, thus preventing disease symptoms from worsening or QoL impairment. Education has been shown to be a protective factor for impaired QoL; however, it is unlikely that this factor directly contributed to disease symptom worsening. Specific factors associated with COVID-19 posed changes such as physical isolation, higher intensity of work load, and reduced income may have contributed to symptom worsening and impaired QoL. These factors have been shown to greatly contribute to mental distress, mainly depression, associated with the COVID-19 pandemic (25). Furthermore, due to increased mental distress during COVID-19, many have sought psychological/psychotherapeutic help and started the use of psychotropic medications (26, 27). Disease-specific factors, such as the duration of illness and available treatment options, were considered as factors that might have contributed to disease symptom worsening and impaired QoL during the COVID-19 pandemic. We also expected that previously-identified risk factors, such as greater age, female sex (28), and depression symptoms would be associated with greater psoriasis symptom worsening and impaired QoL.

We designed a study that allowed us to investigate the factors contributing to disease symptom worsening and impaired QoL in individuals with psoriasis during the COVID-19 pandemic lockdown. We expected that younger age and female sex would be associated with both disease symptom severity and impaired QoL. However, we expected that various aspects of patients' lives, including lower education levels, decreased income, relationships and other previously-identified risk factors (29) would be significantly associated with impaired individuals' psoriasis-related QoL, but not with worsened psoriasis symptoms (30). We expected that clinical markers such as psychotropic medication usage or change in psoriasis treatment regime would be significantly associated with worsening psoriasis condition. Taking into account results from previous studies (31), we also hypothesized that individuals' depression symptom severity would be associated with both worsened clinical symptoms of psoriasis and impaired health-related QoL. Our exploratory aim was to investigate the association of specific depression symptoms with worsened clinical symptoms of psoriasis and impaired disease-related QoL.

## Materials and methods

### Procedure

Adult (over 18 years) subjects with a psoriasis diagnosis were invited to participate in this study and fill in an anonymous online survey. Information about the study and an invitation for participation was provided by the study researchers, sharing information about the study and the link to the online survey to primary care physicians and to a number of psoriasis patient social media groups. Inclusion criteria for the study were: adult (over 18 years old) subjects with a diagnosis of any type of psoriasis at any time during their life. This cross-sectional study was conducted between January and April 2021, during the second COVID-19 lockdown period in Lithuania. The study procedures were approved by the Bioethics Center of the of Lithuanian University of Health Sciences (Approval no. BEC-LSMU (R)-19, January 21, 2021). Before starting the survey, participants had to provide online informed consent to participate in the study by ticking the appropriate answer “agree/disagree.” Of the 306 respondents who accepted the invitation and completed the survey, data from nine surveys were excluded from the final analysis due to not having a confirmed clinical diagnosis of psoriasis. There were no significant differences among the included and excluded subjects in terms of age or sex (*p* > 0.05). Otherwise, there were no missing data in our dataset and the remaining sample of 297 was comprised of the individuals who fully completed the questionnaire. However, the engagement rate for accessing the questionnaire was not monitored.

The minimal study sample size needed to detect a significant difference between the means of two groups of subjects with psoriasis with different disease-related QoL, with 80% power at the 5% level of significance, was calculated to be 186 participants (32).

### Methods

The survey was composed of three parts. The first part of the survey asked individuals for sociodemographic and life-style factor information, evaluating possible triggers and risk factors for exacerbation of psoriasis symptoms. This included respondents' age, marital status, education, work and leisure activities, income and income change during the COVID-19 lockdown. Also included was information about clinical manifestations of psoriasis (confirmation of psoriasis diagnosis, psoriasis-related clinical symptoms, duration of the disease, and treatment methods) and changes in mental health symptoms, and initiation or change in psychotropic medications and/or psychotherapeutic interventions during the COVID-19 lockdown period. The psoriasis symptom worsening was assessed using a single question “During the COVID-19 pandemic (since approximately March 2020), have the symptoms caused by psoriasis changed” with possible answer ranging from “0” “symptoms got better” to “5” “significantly worsened symptoms”. More information about questions provided for the study participants can be found in the Supplementary material.

In the next two parts of the evaluation, two standardized questionnaires were used: the Dermatology Life Quality Index (DLQI) (33) and the Patients' Health Questionnaire-9 (PHQ-9) (34, 35).

#### DLQI

The DLQI questionnaire is the first dermatological QoL questionnaire, published in 1994 (33). This questionnaire is used to measure the impact of most dermatological diseases on the health-related QoL of an affected person. The aim of the use of this questionnaire in the current study was to assess the effect of the severity of psoriasis on the patient's disease-related QoL over the past week. This is a self-reported questionnaire, which consists of 10 short questions that assess the following areas of the patient's life due to their skin condition during the last week: physical symptoms and feelings; daily activity; leisure time; work/school/studies; personal relationships with friends/relatives/partners; and treatment. Each question of the DLQI questionnaire was scored on a four-point Likert scale: very much/yes-3, a lot-2, a little-1, and not at all/not relevant/question unanswered/no-0. The final score of the questionnaire is the sum of the scores of all questions, with a maximum score of 30 points. Over a threshold score of DLQI > 10, individuals are considered to have moderately-to-severely impaired QoL. The higher the final score, the lower the patient's disease-related QoL. The internal consistency of the questionnaire in this sample is considered to be good (Cronbach's alpha 0.895).

#### PHQ-9

The PHQ-9 is a brief self-rated questionnaire, which is part of the PHQ for the assessment of the severity of depressive symptoms during the past 2 weeks. The questionnaire consists of nine items that fit the Diagnostic and Statistical Manual-IV diagnostic criteria for major depressive disorder (34, 35). For each question, one of the four responses should be marked to describe how often the symptom in question has occurred in the last 2 weeks, with each of the nine items scored according to a Likert scale from 0 (“not at all”) to 3 (“nearly every day”). The severity of depressive symptoms was assessed by the sum of the scores of the nine items, and ranges from 0 to 27, where higher scores indicate more severe depressive symptoms. A threshold of ≥10 for the PHQ-9 was considered to indicate an increased risk for depression (35). There was an additional question that asked the respondent to assess how the depressive symptoms were affecting their everyday life, work activities or communication with other people, with self-ratings of: “not difficult at all”, “somewhat difficult”, “very difficult”, and “extremely difficult”. The internal consistency of the scale in this study sample is considered to be good (Cronbach's α 0.901).

### Statistical analysis

The data were analyzed with SPSS Version 27.0.0 (IBM, USA). Mann-Whitney *U* test were used to examine the continuous variables, and chi-square tests were used to test the categorical variables.

The differences in sociodemographic, clinical and life-style factors, and mental distress symptoms reported by individuals who experienced worsened psoriasis symptoms and those who did not experience any change in symptoms were assessed. Mann-Whitney U test were applied to compare PHQ-9 and DLQI scores, and chi-square tests were used for comparisons in terms of sex, family status, education, activity in relation to the COVID-19 lockdown, intensity of workload, income, duration of psoriasis, treatment of psoriasis, the need for changes in psoriasis treatment during the COVID-19 lockdown, and whether they had to seek psychological/psychotherapeutic help. The same comparisons were later performed in individuals who reported impaired disease-related QoL (threshold score of DLQI > 10) vs. individuals with psoriasis who experienced only mild impairment in their health-related QoL (DLQI ≤ 10) during the COVID-19 pandemic. This comparative analysis was conducted in order to investigate possible significant differences between the two groups and identify those variables that might play a role in psoriasis symptom worsening and impaired QoL during the COVID-19 pandemic.

Two separate logistic regression analyses were then performed to investigate associations between sociodemographic factors, life-style factors and mental distress symptoms, and worsened psoriasis symptoms and impaired health-related QoL. The response variable for the first regression model was worsened psoriasis symptoms (classified as 0, “no change or better” or 1, “worsened”), whereas, for the second regression model, the response variable was health-related QoL (classified as 0, “satisfactory QoL (DLQI ≤ 10)” or 1, “moderate-to-severe impairment of QoL (DLQI > 10)”.

At the final stage, we performed logistic regression analyses (stepwise method) to determine whether specific PHQ-9 items predicted psoriasis symptom worsening and impaired QoL more precisely compared to the total score of the scale.

## Results

### Sociodemographic and clinical characteristics

The final sample comprised 297 individuals with psoriasis who completed the survey. Table 1 shows the sociodemographic, clinical and life-style factors, and mental distress characteristics of the individuals overall and by group. The age of the individuals ranged from 18 to 69 years old (M = 34; SD = 10), and the majority were female (*n* = 232; 78.1%). Most of the sample were highly educated, with at least a bachelor's degree (>15 years of studies) (*n* = 173; 58.6%), and with stable or increased income during the pandemic (*n* = 211; 71.1%). A considerable number of individuals reported over 5 years of history of psoriasis (*n* = 239; 80.5%), and most of the participants were receiving topical treatment (*n* = 213; 71.7%) including direct application of topical drugs onto skin rashes (including emollients, moisturizers, vitamin D3 derivatives, retinoids, glucocorticoids, calcineurin inhibitors, anti-interleukin 8 [IL-8] monoclonal antibodies, and coal tar preparations).

**Table 1:** Changes in clinical symptoms of psoriasis and disease-related quality of life during the COVID-19 lockdown in relation to sociodemographic and life-style factors, and mental symptoms.

	**All, *n =* 297 (100%)**	**Psoriasis symptoms improved/ not changed *n =* 141 (47.5%)**	**Psoriasis symptoms worsened *n =* 156 (52.5%)**	**z/χ^2^**	***p***	**DLQI ≤ 10, *n =* 227 (76.4%)**	**DLQI > 10, *n =* 70 (23.6%)**	**z/χ^2^**	***p***
Age, years; median (IQR)	33.0 (27.0–40.0)	33.0 (28.0–39.0)	32.0 (25.3–40.0)	−0.82	0.23	33.0 (27.0–40.0)	31.0 (27.0–40.0)	−0.37	0.71
Sex, n (%)				2.09	0.1672			8.24	0.004
Male	65 (21.9)	36 (25.5)	29 (18.6)			41 (18.1)	24 (34.3)		
Female	232 (78.1)	105 (74.5)	127 (81.4)			186 (81.9)	46 (65.7)		
Family status, n (%)				2.47	0.2971			2.33	0.36
Lives as a couple	230 (77.4)	111 (78.7)	119 (76.3)			177 (78.0)	53 (75.7)		
Single	38 (12.8)	14 (9.9)	24 (15.4)			26 (11.5)	12 (17.1)		
Lives with family members	29 (9.8)	16 (11.3)	13 (8.3)			24 (10.6)	5 (7.1)		
Education, n (%)				3.67	0.4572			3.58	0.47
≤ 10 years	26 (8.8)	8 (5.7)	18 (11.5)			17 (7.5)	9 (12.9)		
11–12 years	35 (11.8)	16 (11.3)	19 (12.2)			26 (11.50)	9 (12.9)		
13–14 years	63 (21.2)	31 (22.0)	32 (20.5)			46 (20.3)	17 (24.3)		
15–16 years	97 (32.7)	50 (35.5)	47 (30.1)			76 (33.5)	21 (30.0)		
> 16 years	76 (25.6)	36 (25.5)	40 (25.6)			62 (27.3)	14 (20.0)		
Activity in relation to the COVID-19 lockdown, n (%)				8.84	0.003			0.69	0.41
Unlimited	123 (41.4)	71 (50.4)	52 (33.3)			97 (42.7)	26 (37.1)		
Limited/self-isolation	174 (59.2)	70 (49.6)	104 (66.7)			130 (57.3)	44 (62.9)		
Intensity of workload, n (%)				1.18	0.29			0.94	0.33
Increased	76 (25.6)	32 (22.7)	44 (28.2)			55 (24.2)	21 (30.0)		
Decreased/not changed	221 (74.4)	109 (77.3)	112 (71.8)			172 (75.8)	49 (70.0)		
Income, n (%)				14.43	0.001			2.98	0.08
Decreased/no income	86 (29.0)	26 (18.4)	60 (38.5)			60 (26.4)	26 (37.1)		
Increased/not changed	211 (71.0)	115 (81.6)	96 (61.5)			167 (73.6)	44 (62.9)		
Duration of psoriasis, n (%)				0.59	0.75			3.88	0.14
< 1 year	17 (5.7)	7 (5.0)	10 (6.4)			15 (6.6)	2 (2.9)		
1–5 years	41 (13.8)	18 (12.8)	23 (14.7)			35 (15.4)	6 (8.6)		
> 5 years	239 (80.5)	116 (82.3)	123 (78.8)			177 (78.0)	62 (88.6)		
Treatment of psoriasis, n (%)				13.34	0.02			2.13	0.83
Combination therapy^a^	15 (5.1)	6 (4.3)	9 (5.8)			11 (4.8)	4 (5.7)		
Topical therapy^b^	213 (71.7)	91 (64.5)	122 (78.2)			165 (72.7)	48 (68.6)		
Systemic therapy^c^	23 (7.7)	12 (8.5)	11 (7.1)			18 (7.9)	5 (7.1)		
Biologic therapy^d^	9 (3.0)	8 (5.7)	1 (0.6)			7 (3.1)	2 (2.9)		
Phototherapy^e^	18 (6.1)	11 (7.8)	7 (4.5)			14 (6.2)	4 (5.7)		
Do not use any treatment	19 (6.4)	13 (9.2)	6 (3.8)			12 (5.3)	7 (10.0)		
Need for changes in psoriasis treatment during the COVID-19 lockdown, n (%)				12.23	< 0.001			5.91	0.02
Yes	59 (19.9)	16 (11.3)	43 (27.6)			38 (16.7)	21 (30.0)		
No	238 (80.1)	125 (88.7)	113 (72.4)			189 (83.3)	49 (70.0)		
Started use of psychotropic medications or increased their doses, n (%)	52 (17.5)	16 (11.3)	36 (23.1)	7.06	0.009	36 (15.9)	16 (22.9)	1.81	0.18
Need to seek psychological/psychotherapeutic help, n (%)	31 (10.4)	10 (7.1)	21 (13.5)	3.21	0.09	19 (8.4)	12 (17.1)	4.41	0.04
**DLQI**									
DLQI, total score; median (IQR)	5.0 (2.0–10.0)	2.0 (1.0–6.5)	7.5 (3.0–13.0)	−3.95	< 0.001	3.0 (1.0–6.0)	15.0 (12.8–19.0)	−8.93	< 0.001
**PHQ-9**									
PHQ-9, total score; median (IQR)	9.0 (4.0–14.0)	5.0 (2.0–10.5)	11.5 (7.0–16.0)	−6.10	< 0.001	7.0 (3.0–13.0)	12.0 (8.0–17.0)	−4.21	< 0.001
PHQ-9 total score ≥ 10	128 (43.1)	38 (27.0)	90 (57.7)	28.54	< 0.001	86 (37.9)	42 (60.0)	10.67	0.001

SD, Standard deviation; z, Mann-Whitney U test; χ^2^, chi-squared test; DLQI, Dermatology Life Quality Index; PHQ-9, Patient Health Questionnaire-9.

a Combination therapy, more than one agent is prescribed;

b Topical therapy, the direct application of topical drugs onto skin rashes (including emollients, moisturizers, vitamin D3 derivatives, retinoids, glucocorticoids, calcineurin inhibitors, anti-interleukin 8 [IL-8] monoclonal antibodies, and coal tar preparations);

c Systemic therapy, oral and injected medications that work throughout the entire body (including methotrexate, cyclosporine, acitretin, mycophenolic acid, hydroxyurea, 6-thioguanine, and other *systemic* agents);

d Biologic therapy, biologic drugs designed to act on specific immune system targets, as TNFα (including etanercept, infliximab, adalimumab and certolizumab), IL-12/IL-23 (including ustekinumab), IL-17 (including secukinumab, ixekizumab and brodalumab) and IL-23 (including guselkumab, tildrakizumab and risankizumab);

e Phototherapy, *treatment* that *uses ultraviolet rays* with in the *UVA* and *UVB* spectrum.

A total of 52 (17.5%) individuals reported starting use of psychotropic medications or increasing their dosage and 31 (10.4%) individuals reported seeking psychological/psychotherapeutic help during the COVID-19 pandemic. Almost half of the individuals responding to the survey reported significant depression symptoms (PHQ-9 ≥ 10, *n* = 128; 43.1%). Impaired disease-related QoL was reported by a smaller number of individuals (DLQI > 10, *n* = 70; 23.6%).

### Worsening of psoriasis symptoms

Decreased activity in relation to the COVID-19 lockdown was found among those who reported worsened psoriasis symptoms [χ^2^(1, *N* = 297) = 8.84, *p* = 0.003; *N* = 156], with a greater proportion of respondents with limited activity or self-isolation (*n* = 104, 66.7%) reporting worsened symptoms compared to those with unlimited activity (*n* = 52; 33.3%).

Decreased income was also among the factors related to worsened psoriasis symptoms [χ^2^(1, *N* = 297) = 14.43, *p* = 0.001], together with the need for changes in psoriasis treatment during the COVID-19 lockdown [χ^2^(1, *N* = 297) = 12.23, p < 0.001], and starting the use of psychotropic medications or increasing their dosage [χ^2^(1, *N* = 297) = 7.06, *p* = 0.009].

The median score on the PHQ-9 was 9.0 (range: 4.0–14.0), with individuals with worsened psoriasis symptoms (median 11.5; range: 7.0–16.0 showing significantly higher values than those who experienced no change in their symptoms (median 5; range: 2.0–10.5), [z(295) =-6.10, p < 0.001, d = 0.76].

The DLQI scores were also significantly higher in individuals with worsened psoriasis symptoms (median 7.5; range: 3.0–13.0) in comparison to individuals who reported no such changes (median 2.0; range 1.0–6.5), [z(295) =-3.95, p < 0.001, d = 0.42].

### Impaired health-related QoL

A sex difference was found among those who reported impaired QoL [χ2(1, *N* = 297) = 8.24, p < 0.004; *N* = 70], with a greater proportion of female (*n* = 46; 65.7%) than male (*n* = 24, 34.3%) participants reporting QoL impairment.

The group with impaired disease-related QoL (based on the DLQI threshold score of >10) comprised a higher number of individuals who needed to change psoriasis treatment during the COVID-19 lockdown [χ^2^(1, *N* = 297) = 5.91, p < 0.015], needed to seek psychological/psychotherapeutic help [17.1 vs. 8.4%, χ^2^(1, *N* = 297) = 4.41, *p* = 0.036], and who had a higher severity of depressive symptoms [PHQ-9 total score (median 12.0; range 8.0–17.0) vs. (median 7.0; range: 3.0–13.0), z(295) = −4.21, p < 0.001, d = 0.50].

### Predictors of worsened psoriasis symptoms

Logistic regression to identify predictors of worsened psoriasis symptoms (classified as 0, “no change” or 1, “worsened”) included sex (1, “male”; 2, “female”), age, activity in relation to the COVID-19 lockdown, income, treatment of psoriasis, need for changes to psoriasis treatment during the COVID-19 lockdown, starting use of psychotropic medications or increasing their doses, and PHQ-9 (Table 2). The strongest predictor of worsened symptoms was the need for changes in psoriasis treatment during the COVID-19 lockdown, with an odds ratio (OR) of 2.73 (95% CI 1.37–5.44, *p* = 0.004). The probability of symptom worsening doubled with decreased income in comparison to stable or increased income during the COVID-19 pandemic (OR = 2.33, 95% CI 1.30–4.17, *p* = 0.004). Depression symptoms were also significantly associated with worsening of psoriasis symptoms during the COVID-19 lockdown (OR = 1.10, 95% CI 1.05–1.14, p < 0.001).

**Table 2:** Multivariate regression analysis of factors associated with subjectively worsened psoriasis symptoms, including both sociodemographic factors and severity of depressive symptoms (PHQ-9 total score).

**Factors**	**R^2^ = 0.241**	**OR**	**95% CI**	***p***
Sex		1.24	0.66–2.30	0.50
Age		0.99	0.97–1.02	0.76
Activity in relation to the COVID-19 lockdown		1.43	0.85–2.40	0.181
Income		2.33	1.30–4.17	0.004
Treatment of psoriasis		0.87	0.74–1.03	0.12
Need for changes in psoriasis treatment during the COVID-19 lockdown		2.73	1.37–5.44	0.004
Started use of psychotropic medications or increased their doses		1.25	0.60–2.60	0.55
PHQ-9		1.10	1.05–1.14	< 0.001

95% CI, confidence interval; OR, odds ratio; PHQ-9, Patient Health Questionnaire-9; Sex (male, 1/female, 2); Age (years); Activity in relation to the COVID-19 lockdown (Unlimited, 0/ Limited/self-isolation, 1); Income (Increased/not changed, 0/ Decreased/no income, 1); Need for changes in psoriasis treatment during the COVID-19 lockdown (No, 0/ Yes, 1); Started use of psychotropic medications or increased their doses (No, 0/ Yes, 1).

A stepwise logistic regression model for specific depression items showed that the PHQ-9 item 2 “feeling down, depressed, or hopeless” predicted symptom worsening even better than the sum score of the PHQ-9, increasing the R2 by 0.282 (OR = 2.22, 95% CI 1.65–2.99, p < 0.001) (Supplementary Table 1S).

### Predictors of impaired QoL

Logistic regression on disease-related QoL (classified as 0 “DLQI ≤ 10” or 1, “DLQI > 10”) included sex (2, “male”; 1 “female”), age, the need for changes in psoriasis treatment during the COVID-19 lockdown, need to seek psychological/psychotherapeutic help, psoriasis symptoms worsening, and PHQ-9 (Table 3). The strongest predictor was male sex, which improved the chances of impaired QoL during the COVID-19 lockdown in comparison to female sex (OR = 3.35, 95% CI 1.70–6.59, p < 0.001). The probability of being attributed to the group with impaired QoL was also tripled if the individual experienced worsened psoriasis symptoms during the COVID-19 lockdown (OR = 3.22, 95% CI 1.66–6.24, p < 0.001). Depression also contributed to the probability of impaired QoL (OR = 1.07, 95% CI 1.02–1.16, *p* = 0.008).

**Table 3:** Multivariate regression analysis of factors associated with impaired disease-related quality of life (DLQI > 10) in patients with psoriasis, including both sociodemographic factors and severity of depressive symptoms (PHQ-9 total score).

**Factors**	**R^2^ = 0.203**	**OR**	**95% CI**	***p***
Sex		3.35	1.70–6.59	< 0.001
Age		0.99	0.97–1.03	0.90
Need for changes in psoriasis treatment during the COVID-19 lockdown		1.53	0.78–2.99	0.22
Need to seek psychological/ psychotherapeutic help		1.58	0.67–3.73	0.30
Psoriasis symptoms worsened		3.22	1.66–6.24	< 0.001
PHQ-9		1.07	1.02–1.16	0.008

95% CI, confidence interval; OR, odds ratio; PHQ-9, Patient Health Questionnaire-9; Sex (female, 1/male, 2); Age (years); Need for changes in psoriasis treatment during the COVID-19 lockdown (No, 0/ Yes, 1); Need to seek psychological/psychotherapeutic help (No, 0/ Yes, 1); Psoriasis symptoms worsened (no change or better, 0/ worsened, 1).

A stepwise logistic regression model for specific depression items showed that the PHQ-9 item 9 “Thoughts that you would be better off dead, or of hurting yourself in some way” predicted impaired QoL better than the sum score of the PHQ-9, increasing the R2 by 0.231 (OR = 1.58, 95% CI 1.12–2.24, *p* = 0.010) (Supplementary Table 2S).

## Discussion

In the current study, we aimed to investigate whether sociodemographic and COVID-19 lockdown lifestyle factors affected changes in clinical symptoms of psoriasis and psoriasis-related QoL, and to evaluate the contribution of specific mental symptoms, such as depressive symptoms, in this process. We found that the need for changes in psoriasis treatment during the COVID-19 lockdown, decreased income, and depression symptoms were the strongest predictors of psoriasis symptom worsening. Psoriasis-related QoL during the COVID-19 lockdown was associated with male sex, psoriasis symptoms worsening, and depression symptoms. Specific symptoms of depression, such as thoughts of feeling down, depressed or hopeless, were associated with psoriasis symptom worsening, while suicidal thoughts were associated with impaired psoriasis-related QoL.

The main finding in this study was that the predictors for worsened psoriasis symptoms during the COVID-19 lockdown were limited to decreased income and the need for changes in psoriasis treatment, starting use of psychotropic medications or changing their doses, and higher severity of depressive symptoms (36, 37). A similar web-based survey conducted in China confirms our findings, revealing a similar conclusion; as in this study, exacerbation of psoriasis was associated with outdoor activity restriction and income loss (38). Some people with psoriasis had stopped or changed their treatment of psoriasis during the COVID-19 lockdown. The leading reasons were perceived stress, fear, worry, depression, and anxiety about the risk of being infected with COVID-19. These data indicate a burden due to the COVID-19 pandemic in people with psoriasis; worsening psoriasis is common and is associated with poor mental health (39). Furthermore, studies show that, in individuals with psoriasis, depression is associated with increased risk of myocardial infarction, stroke and cardiovascular death, especially during acute depression (40).

Overall, the strongest predictor of worsened psoriasis symptoms during the COVID-19 lockdown was limited access to health care (38), which caused difficulties in continuous treatment for patients with chronic diseases, non-adherence to treatment and adverse health outcomes. We observed a consequent need to change psoriasis treatment in the current study sample. Only 8% of individuals in our study sample were receiving systemic psoriasis treatment during COVID-19 period. However, it is estimated that around 17% of individuals experiencing psoriasis symptoms ranging from moderate to severe, require systemic treatment (41). No possibilities to initiate such treatment due to lockdown might have fueled individuals' with psoriasis symptoms of depression and impaired QoL.

Next, we tested factors associated with psoriasis-related QoL. Our findings showed that predictors for impaired psoriasis-related QoL were male sex, worsened psoriasis symptoms, and higher severity of depressive symptoms.

A similar self-administered web-based questionnaire was distributed through social media by Yeye Guo et al. (7). Authors found that isolation, income loss and unemployment were associated with impaired health-related QoL in patients with skin diseases during the COVID-19 pandemic (7). Also, outdoor activity restriction was significantly associated with anxiety, depression and impaired QoL (7). Besides depression being one of the strongest predictors of impaired QoL, our study does not confirm the findings of Guo et al. (7). This discrepancy may be explained by the fact that Guo et al. (7) did not use psoriasis symptom worsening in their prediction models. It is possible that the pathway between impaired psoriasis-related QoL and reduced income and isolation is mediated *via* symptom worsening. Thus, symptom worsening might be a mediating factor in this process; however, investigation of a mediation model was beyond the scope of our study.

Contrary to our hypothesis, male, but not female, sex contributed to impaired psoriasis-related QoL. Results in the scientific literature on the role of sex differences in psoriasis-related QoL are somewhat contradictory, with some studies showing no sex differences (21, 42), some reporting lower QoL for females in comparison to males (43, 44), and some suggesting the opposite (45). However, in our study sample, we had more males than females (21.5 vs. 10.3%) who were not receiving treatment for psoriasis, and more males than females (13.8 vs. 4.3%) who were living alone during the COVID-19 pandemic. These factors may have influenced the relationship between sex and psoriasis-related QoL.

Besides well spotted factors in prediction modeling for both psoriasis symptom worsening and impaired QoL, several other characteristics should be considered in future studies investigating aforementioned associations. COVID-19 period was marked not only with increased numbers of depression but also with anxiety disorders (46, 47) accompanied with certain cognitive difficulties attributed to the COVID-19 infection (48) or, such as inflexible thinking style, to personality features (49). Several other characteristics, such as stigmatization (50, 51) and alexithymia (52–57) have been shown to predict psoriasis symptom worsening as well as impaired OoL. Unfortunately, due to the brevity of our survey, we have not included these factors which might have also contributed to the prediction of symptom worsening and impaired QoL in our study design.

Lastly, we observed that some of the individual questions of the PHQ-9 were even better than the whole questionnaire in prediction modeling for both psoriasis symptom worsening and impaired psoriasis-related QoL. The question about “Feeling down, depressed, or hopeless” was associated with psoriasis symptom worsening, while “Thoughts that you would be better off dead, or of hurting yourself in some way” was associated with impaired psoriasis-related QoL. The question on being depressed summarizes a cardinal feature of the mental disorder, which other studies have also shown to have good psychometric characteristics in its prediction (58). As expected, psoriasis symptom worsening was greatly affected by depression symptoms, and thus the question on the particular experience of being depressed predicted the probability of symptom worsening. On the other hand, the question regarding suicidal thoughts contributed most to impaired psoriasis-related QoL, besides male sex and psoriasis symptom worsening. This symptom is common in depression, with a higher prevalence in male than in female. Since male sex was one of the main contributors to impaired psoriasis-related QoL, and more males than female experienced suicidal ideation (12.3 vs. 3.4%, based on the PHQ-9 question) more than half of the days, we believe that question on suicidal ideation added more to the model than indicating a general feeling of depression, which was common in both males and females.

The limitations of the study included the selection bias associated with online surveys and recall bias of patient-reported outcomes. Thus the reader has to take into account that we assessed subjectively experienced symptom worsening rather than the objective clinical documentation of psoriasis exacerbation. Furthermore, to identify any treatment changes we used generally phrased item “Did you have your medical treatment of psoriasis changed during the COVID-19 pandemic”. This phrasing precludes identifying whether treatment change was related to the pandemic (e.g., restricted access to care) or a result of symptom changes. We acknowledge, that in general there is a large association between symptom worsening and the need to change the treatment. If symptoms worsen then dermatologist might prescribe different medication that might work better. Existing restriction in accessing such care might have contributed to the need for psoriasis treatment change. Since “change in psoriasis treatment” is one of the main predictors in the model, careful understanding of our item phrasing is important not to overstate our study results.

It might be also relevant to consider the effect of vaccination on psoriasis symptom worsening. Vaccination is not common trigger for psoriasis symptoms exacerbation (59), but there are some reports of psoriasis symptoms worsening after vaccination for influenza, pneumococcal pneumonia, and yellow fever (60). New onset and exacerbation of psoriasis were reported in the systemic review and case series documenting new diagnosis of psoriasis or psoriasis exacerbation after at least one dose of any COVID-19 vaccine (61, 62). However, COVID-19 vaccination in Lithuania has actively started in Jan 2021 and lasted until the end of this study (Apr 2021) with 24.7% of Lithuanian population receiving the first dose of the vaccine. By the end of the study data collection only 10.8 % of Lithuanian were fully vaccinated (63). Unfortunately, our study has no data on vaccination during the period of data collection.

Furthermore, our study sample was relatively small and consisted of individuals in their early thirties. Thus, the results may not represent geriatric patients, who were less accessible *via* the internet or social media or those with more severe psoriasis conditions (72 % of individuals in our study were using topical agents). The engagement rate was not monitored precluding information on actual interest in participating in the study.

Hopefully, taking our research into consideration, several health and research practices could be implemented. In our study were able to identify specific modifiable and non-modifiable factors related with both psoriasis symptom worsening and impaired QoL in individuals with psoriasis during COVID-19 pandemic. Along with other well-known risk factors (such as alcohol use, smoking, anxiety and alexithymia), the factors we identified could be used for targeted prevention and intervention. Furthermore, based on our analysis on specific depression symptoms, spotting these, could also be used for future practices to detect individuals vulnerable to symptom worsening and impaired QoL.

## Conclusion

More than half of psoriasis patients reported subjectively worsened psoriasis symptoms during the COVID-19 lockdown period, and one quarter were evaluated as having impaired psoriasis-related QoL.

Worsened psoriasis symptoms during the COVID-19 lockdown are associated with decreased income, psoriasis treatment changes and depression symptoms. QoL impairment is associated with male sex, psoriasis symptom worsening and depression. Specific depression symptoms may have contributed more to symptom worsening and impaired QoL than the depressive symptomatology as a whole.

## Data availability statement

The raw data supporting the conclusions of this article will be made available by the authors, without undue reservation.

## Ethics statement

The study and it's consent procedures were approved by Bioethics Center of Lithuanian University of Health Sciences [Approval no. BEC-LSMU (R)-19, January 21, 2021]. The patients/participants provided their written informed consent to participate in this study.

## Author contributions

JB and VS: designed the study. MS: collected and analyzed the data. AP: statistical analyses. JB, MS, AP, and VS: drafted and edited the manuscript. All authors contributed to the manuscript and approved the final version.
