# Supplementary material for: Factors associated with worsened clinical symptoms of psoriasis and disease-related quality of life during the COVID-19 lockdown: A cross-sectional study
Source: Front Med (Lausanne). 2023 Jan 10;9:1027853. doi: 10.3389/fmed.2022.1027853 (PMC9871456; doi:10.3389/fmed.2022.1027853)
Supplement: Supplementary file 1 [file Table_1.docx]

Supplementary Material

**Table 1S**

Stepwise analysis of factors associated with subjectively worsened psoriasis symptoms

|  | R² = 0.282 | OR | 95% CI | p |
| --- | --- | --- | --- | --- |
| Sex |  | 1.21 | 0.64–2.29 | 0.560 |
| Age |  | 0.99 | 0.97–1.02 | 0.661 |
| Activity in relation to the COVID-19 lockdown |  | 1.42 | 0.84–2.40 | 0.195 |
| Income |  | 2.23 | 1.23–4.04 | 0.008 |
| Treatment of psoriasis |  | 0.87 | 0.73–1.03 | 0.105 |
| Need for changes in psoriasis treatment during the COVID-19 lockdown |  | 2.54 | 1.26–5.12 | 0.004 |
| Started use of psychotropic medications or increased their doses |  | 1.29 | 0.62–2.67 | 0.497 |
| PHQ-9 item: Feeling down, depressed, or hopeless |  | 2.22 | 1.65–2.99 | <0.001 |

Notes: 95% CI, confidence interval; OR, odds ratio; PHQ-9, Patient Health Questionnaire-9; Sex (male, 1/female, 2); Age (years); Activity in relation to the COVID-19 lockdown (Unlimited, 0/ Limited/self-isolation, 1); Income (Increased/not changed, 0/ Decreased/no income, 1); Need for changes in psoriasis treatment during the COVID-19 lockdown (No, 0/ Yes, 1); Started use of psychotropic medications or increased their doses (No, 0/ Yes, 1)

**Table 2S**

Stepwise analysis of factors associated with impaired disease-related quality of life (DLQI > 10)

|  | R^2^ = 0.231 | OR | 95% CI | p |
| --- | --- | --- | --- | --- |
| Sex |  | 2.86 | 0.19–0.72 | 0.002 |
| Age |  | 0.99 | 0.97–1.03 | 0.844 |
| Need for changes in psoriasis treatment during the COVID-19 lockdown |  | 1.58 | 0.79–3.13 | 0.193 |
| Need to seek psychological/psychotherapeutic help |  | 1.60 | 0.68–3.79 | 0.284 |
| Psoriasis symptoms worsened |  | 3.51 | 1.81–6.78 | <0.001 |
| PHQ-9 item: Thoughts that you would be better off dead, or of hurting yourself in some way |  | 1.58 | 1.12–2.24 | 0.010 |

Notes: 95% CI, confidence interval; OR, odds ratio; PHQ-9, Patient Health Questionnaire-9; Sex (female, 1/male, 2); Age (years); Need for changes in psoriasis treatment during the COVID-19 lockdown (No, 0/ Yes, 1); Need to seek psychological/psychotherapeutic help (No, 0/ Yes, 1); Psoriasis symptoms worsened (no change or better, 0/ worsened, 1)
