# Supplementary material for: Factors associated with worsened clinical symptoms of psoriasis and disease-related quality of life during the COVID-19 lockdown: A cross-sectional study
Source: Front Med (Lausanne). 2023 Jan 10;9:1027853. doi: 10.3389/fmed.2022.1027853 (PMC9871456; doi:10.3389/fmed.2022.1027853)
Supplement: Supplementary file 2 [file Data_Sheet_1.docx]

Supplementary Material

***Socio-demographic questionnaire***

**1. What sex were you assigned at birth (on your original birth certificate)?**

Male

Female

**2. How old are you? (years)**

**3. What is your relationship status?**

 Single

 Living as a couple

Living with family members

**4. What is your highest level of education (how many years did you study in total)?**

10 years or less

11-12 years old

13-14 years old

15-16 years old

More than 16 years

**5. Are you currently limited to leave your residence for outside activity?**

Unlimited

Limited/self-isolation

**7. Has your income changed during the COVID-19 pandemic (approximately since March 2020)?**

Increased/ not changed

Decreased/ no income

**8. How long have you had psoriasis?**

<1 year old

1-5 years

>5 years old

**9. How do you treat psoriasis?**

 **Combination therapy**, more than one agent is prescribed.

 **Topical therapy** (the direct application of topical drugs onto skin rashes (including emollients, moisturisers, vitamin D3 derivatives, retinoids, glucocorticoids, calcineurin inhibitors, anti-interleukin 8 [IL-8] monoclonal antibodies, and coal tar preparations); Systemic treatment (Methotrexate, Cyclosporine, Acitretin)

 **Systemic therapy** (oral and injected medications that work throughout the entire body (including methotrexate,cyclosporine, acitretin, mycophenolic acid, hydroxyurea, 6-thioguanine, and other systemic agents);

 **Biologic therapy** (biologic drugs designed to act on specific immune system targets, as TNFα (including etanercept, infliximab, adalimumab and certolizumab), IL-12/IL-23 (including ustekinumab), IL-17 (including secukinumab, ixekizumab and brodalumab) and IL-23 (including guselkumab, tildrakizumab and risankizumab);

 **Phototherapy** (treatment that usesultraviolet rays with in the UVA and UVB spectrum)

 I have not received any treatment

 Other (please describe below):

**11. Did you have your medical treatment of psoriasis changed during the COVID-19 pandemic?**

No

Yes

**12. During the COVID-19 pandemic (since approximately March 2020), have the symptoms caused by psoriasis changed?**

Much improved

Moderately improved

Didn't change

Moderately worsened

It got really bad

**16. During the COVID-19 pandemic, have you started taking psychotropic medications (sedatives, hypnotics, antidepressants, antipsychotic medications) or increased their dosage (if you started taking them before the COVID-19 pandemic)?**

No

Yes

**17. During the COVID-19 pandemic, did seek psychological/psychotherapeutic help due to a worsened mental state?**

No

Yes
